# Sensing and regulation of cell volume – we know so much and yet understand so little: TRPV4 as a sensor of volume changes but possibly without a volume-regulatory role?

**DOI:** 10.1080/19336950.2018.1438009

**Published:** 2018-02-09

**Authors:** Trine L. Toft-Bertelsen, Brian R. Larsen, Nanna MacAulay

**Affiliations:** Department of Neuroscience, University of Copenhagen, Copenhagen, Denmark

**Keywords:** aquaporins, osmo-sensing, volume regulation, volume-sensitive ion channels, transient receptor potential vanilloid 4 channel, TRPV4

## Abstract

Cellular volume changes lead to initiation of cell volume regulatory events, the molecular identity of which remains unresolved. We here discuss experimental challenges associated with investigation of volume regulation during application of large, non-physiological osmotic gradients. The TRPV4 ion channel responds to volume increase irrespectively of the molecular mechanism underlying cell swelling, and is thus considered a sensor of volume changes. Evidence pointing towards the involvement of TRPV4 in subsequent volume regulatory mechanisms is intriguing, yet far from conclusive. We here present an experimental setting with astrocytic cell swelling in the absence of externally applied osmotic gradients, and the lack of evidence for involvement of TRPV4 in this regulatory volume response. Our aim with these new data and the preceding discussion is to stimulate further experimental effort in this area of research to clarify the role of TRPV4 and other channels and transporters in regulatory volume responses.

## Introduction

Maintenance of cell volume is a homeostatic imperative for cells of most origins [1] and cellular responses suited to restore cell volume are set in motion in response to conditions causing cell swelling or shrinkage. Despite the widespread phenomenon and the numerous situations in which cell and tissue volume is compromised during physiology and pathophysiology, the molecular mechanisms governing cell volume homeostasis remain disputed and/or undefined. While many of the suggested volume regulatory (transport) mechanisms appear to sense volume changes, rather than aberrant changes in surrounding osmotic pressure, a few bona-fide osmo-sensing channels and transporters, i.e. BetP, ProP and OpuA have been discovered [2–6]. These membrane proteins sense changes in the osmolarity of the surrounding fluid via alterations in membrane properties/protein-lipid interactions and/or by changes in the intracellular K^+^ concentration, although direct effects via changes in the hydration state of the proteins cannot be excluded [7]. Despite the fact that abruptly arising, large osmotic challenges rarely, if ever, occur within mammalian tissue, a convenient, and therefore common, manner of investigating volume regulatory responses is by introduction of excessive non-physiological osmotic challenges of up to 250 mOsm [8–16]. It is, in addition, not unusual that hyposmotic challenges of this magnitude are experimentally obtained by a dilution of the physiological test solution with distilled water [13–15,17]. Although this experimental approach is intended to simply change the osmotic pressure, the ionic strength will be diminished. This dilution thus causes drastic changes within the ionic equilibrium potentials, associated with a shift in the membrane potential and, in addition, the driving forces for a range of ion channels and transporters. These side effects are predicted to introduce serious confounding elements into the experimental set-up and may thus skew the outcome. We recommend that mannitol is included in the control solution (equiosmolar replacement of NaCl) and its subsequent removal will thus reduce the osmolarity without affecting the ionic strength [9,18,19].

**Figure 1.**
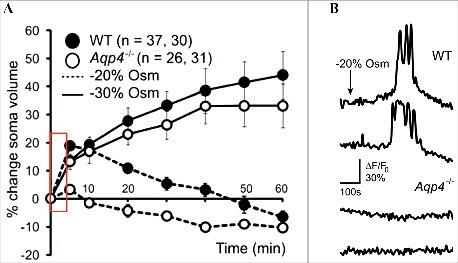
Contribution of AQP4 to astrocytic swelling and Ca^2+^ signaling. A: Cortical slices from wildtype (filled symbols) and AQP4-deficient (open symbols) mice were exposed to a hyposmotic gradient (−20%, 60 mOsm (dashed lines)). Such osmotic challenge induced astrocytic swelling in slices from wildtype mice while these were non-detectable in slices from AQP4-deficient mice (marked with a red box), monitored with two-photon imaging of slices loaded with Texas red hydrazide. Verification of selective dye uptake in astrocytes was confirmed by two-photon imaging of slices from mice expressing GFP in astrocytes (*Glt-1*-EGFP BAC transgenic mice). A larger gradient of −30%, 90 mOsm (solid lines) induced similar swelling in slices from both strains of mice. B: Astrocytes from wildtype (*top panel*) and AQP4-deficient (*lower panel*) mice were loaded with Rhod2 AM and exposed to a hyposmotic gradient of −20% mOsm. Ca^2+^ dynamics were monitored ∼200 sec after introduction of the osmotic challenge (and the equivalent swelling marked with the red box in panel A). At this time point, wildtype astrocytes displayed Ca^2+^ spikes while AQP4-deficient astrocytes did not, presumably due to their absent (or limited) cell swelling. Modified from [20] with permission.

**Figure 2.**
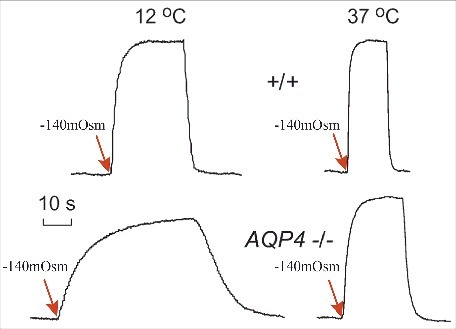
Characterization of water transport in astrocytes from wildtype and AQP4-deficient mice. Primary culture of astrocytes from wildtype mice (*top panel*) or APQ4-deficient mice (*lower panel*) were faced with a hyposmotic challenge of −140 mOsm (marked with an arrow) as obtained by 1:1 dilution of PBS with distilled water. The cellular swelling was monitored at 12°C (*left*) or 37°C (*right*) by the calcein quenching method. Modified from [15] with permission.

A range of studies imply a requirement for aquaporins (AQPs) for activation of volume-sensitive ion channels, and possibly regulatory events, to take place [10,20,21] (Fig. 1). However, as AQPs are required to translate an abruptly introduced osmotic challenge into swift cell volume changes, the non-physiological experimental approach of introducing large osmotic gradients favors such conclusions [8,9,19,22]. In most physiological settings, the buildup of osmotic gradients will necessarily occur gradually with increased transmembrane transport activity and will represent the rate-limiting factor for cell swelling – rather than the osmotic water permeability (and hence AQP expression) of the cell membrane. Of interest, with a few exceptions such as pyramidal neurons [23] and stomach epithelium, the majority of cellular structures are rather water permeable even in the absence of AQPs, most likely via the multitude of water permeable cotransporters and uniporters present in these cells [24–26]. Striking examples are the AQP4-expressing astrocytes, the water permeability of which is reduced by only 50% (at 37˚C) upon AQP4 knock-out [15] or knock-down [16]. As these cells are extremely water permeable to begin with, a 50% reduction of their osmotic water permeability still leaves them with the ability to equilibrate with a sudden osmotic challenge of −140 mOsm well within a few seconds, at the physiological temperature of 37˚C (Fig. 2). It follows that a physiologically relevant gradual buildup of an osmotic gradient (e.g. 1 mOsm at a time) via altered transport/channel activity will be instantly equilibrated – whether AQP4 is expressed or not. This phenomenon was illustrated in an experimental setting with astrocytic cell swelling in the absence of an introduced osmotic challenge: During neuronal activity in murine hippocampal slices (achieved by electric stimulation), the extracellular space (ECS) shrinks, mainly due to astrocytic cell swelling [27,28]. Genetic deletion of AQP4 did not prevent, or even reduce, this cell swelling [21], illustrating that the osmotic water permeability of the astrocytic cell membrane does not represent the rate-limiting step during astrocytic cell swelling (at least under these experimental conditions). We later demonstrated that the stimulus-induced astrocytic cell swelling relies on cotransporter-mediated water transport [27].

## Cell volume regulation

For cells to maintain their volume when faced with a stressor causing alterations in cell volume, their cellular response depends on a volume sensor coupled to a downstream mechanism causing transmembraneous shift of osmotic particles (either as separate entities or residing within one molecular transducer). Volume regulation most likely does not rely on one set of mechanisms but is rather predicted to depend on the molecular make-up of each cell type. A range of cotransport mechanisms have been indicated in cell volume regulation, with the Na^+^/K^+^/2Cl^−^ cotransporter and the Na^+^/H^+^ exchanger as prominent inducers of cell swelling following osmotically induced cell shrinkage and, oppositely, the K^+^/Cl^−^ cotransporter as an inducer of cell shrinkage upon osmotically induced cell swelling (for review, see [29-32]). An intricate regulatory pathway involving cell volume-sensitive kinases of the SPAK/WNK families leads to (de)phosphorylation of the abovementioned transporters, which in turn activates or inhibits the relevant set of transporters [33,34] (for review, see [31]) to promote the appropriate regulatory volume response. These kinases may be sensitive to cell volume changes themselves and/or to the altered intracellular Cl^−^ concentration coming about by the transmembraneous shift of water during a large osmotic challenge [35]. An alternative mechanism involved in acute volume regulation following osmotically-induced cell swelling involves a concerted activity of plasma membrane K^+^ and Cl^−^ channels. This channel activity results in loss of KCl across the plasma membrane with a proposed associated directional water flow. It must be emphasized that this paradigm exclusively will work provided that both conductive pathways coexist and the electrochemical driving forces promote efflux of both ions: The principle of electroneutrality states that an equal amount of positive and negative charges must exist at either side of the membrane (with a miniscule excess of negative charges intracellularly to provide the negative membrane potential), as the lipid bilayer cannot sustain the large voltages that would occur given a millimolar excess of either positive or negative charges on one side of the membrane [36]. It is thus inconceivable that a buildup of an osmotic gradient of several mOsm (required for osmotically-induced water transport) could occur by the activity of a K^+^ (or Cl^−^ or Na^+^) channel alone, although promoted as a feasible manner of driving water movement [37–41]. In the central nervous system, release of several millimolar K^+^ into the ECS (to provide the required driving force for osmotic water efflux) would lead to neuronal depolarization and enhanced excitability. Such a mechanism may thus not be a viable manner of cell volume regulation in the cell structures of the central nervous system.

The activity of a range of different ion channels is modulated by altered cell volume, prominently so both K^+^ channels [42–45] and Cl^−^ channels [46–50] (extensively reviewed in [51,52]). This swelling-induced activation of K^+^ and Cl^−^ channels can occur as a direct sensing of altered cell volume or, alternatively, secondarily to swelling-induced intracellular Ca^2+^ transients. Such Ca^2+^ dynamics may lead to activation of ion channels such as Ca^2+^-dependent K^+^ channels [53,54] and/or initiation of cell signaling pathways with downstream activation of other transport mechanisms effectuating cell volume regulation. However, the linkage between cellular modulators that cause the volume-mediated Ca^2+^ dynamics and molecular pathways effectuating the volume regulatory response remains unresolved. Several volume-/stretch-sensitive Ca^2+^ -permeable channels have been identified [55–61], one of which is the *t*ransient *r*eceptor *p*otential *v*anilloid 4 (TRPV4) ion channel. TRPV4, also known as the *v*anilloid *r*eceptor related and *o*smotically *a*ctivated *c*hannel (VR-OAC [62]) or the *O*SM9-like *t*ransient *r*eceptor *p*otential *c*hannel, member 4 (OTRPC4 [62,63]), is a nonselective cation channel expressed in many cell types: i.e. Müller cells, astrocytes, in neurons of the circumventricular organs, in mechanosensitive neurons of the mammalian inner ear hair cells, in the epithelia of the trachea, oviduct, lung and cochlea, in the airway, blood vessels, and in certain segments of the kidneys [62–70], many of which are key mechanosensory and osmosensory tissues.

## TRPV4 – an ion channel sensitive to abrupt volume changes

TRPV4 is a cation-permeable ion channel originally described as an osmo-sensor due to its activation by large hyposmotic challenges [63,71]. With such externally applied osmotic challenges, co-expression of an(y) AQP promotes the fast rate of osmotically-induced cell swelling leading to TRPV4 activation in astrocytes, Müller cells, and TRPV4-expressing oocytes [8,9,19,22]. However, TRPV4 activation readily occurred by cell swelling induced by cotransport of water *in the absence* of an externally applied osmotic challenge and presence of an AQP [9] and TRPV4 is therefore a genuine sensor of abrupt volume changes irrespective of the origin of the cell swelling. This observation thus promotes the term ‘volume-sensitive’ rather than ‘osmo-sensitive’ [9]. Nevertheless, the structural determinant underlying the repeatedly observed coupling of cell swelling to TRPV4 channel opening [8–11,19,22,62,63] remains unresolved.

With its robust activation upon cell volume increase, TRPV4 has been proposed as a candidate to translate cell swelling to cell volume regulation [10,19,54,72-78] via its Ca^2+^ permeability, which places it in a favorable position as a main conductor of the Ca^2+^ influx required for volume regulatory mechanisms in some settings. The studies addressing the role of TRPV4 in regulatory volume responses were all performed by introduction of a non-physiologically large hyposmotic challenge, which is likely to introduce experimental confounders that may complicate the identification of the volume sensor and/or the molecular mechanisms effectuating the regulatory volume decrease. Of note, as TRPV4-mediated Ca^2+^ influx, in itself, cannot generate regulatory volume decrease, TRPV4 activity must necessarily couple to downstream effectors, currently unidentified, which then promote the loss of electrolytes and water.

## Contribution of TRPV4 to cell volume regulation during physiological relevant events

**Figure 3.**
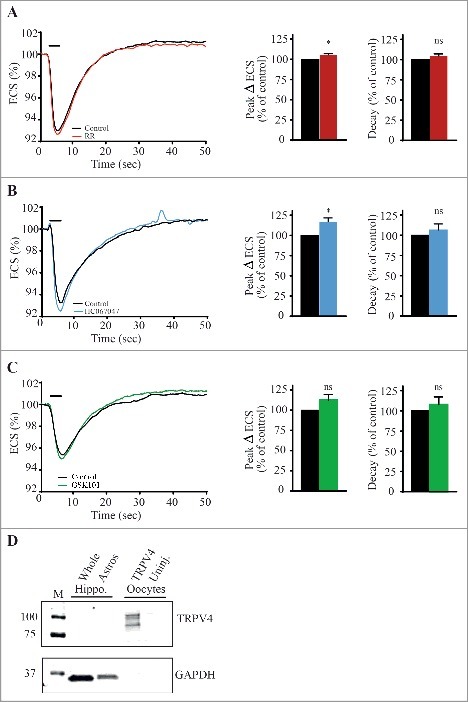
TRPV4 plays a minor, if any, role in regulating astrocyte volume during stimulus-evoked ECS shrinkage in acute hippocampal slices from rats. Ion-sensitive microelectrodes were employed to measure the relative size of the ECS upon addition of 1.5 mM TMA^+^ to the test solution. A-C, left: Representative recordings of the relative ECS obtained in CA1 (stratum radiatum) upon electrical stimulation (20 Hz; indicated by black bar) from slices treated with RR (A), HC067047 (B) or GSK101 (C). The black traces illustrate the control condition and the colored traces the one obtained in the presence of either antagonists or agonist. A-C, right: Normalized data on the peak amplitude, and decay constants. D: Representative Western blot illustrating the expression of TRPV4 in whole hippocampal tissue homogenate (20 μg total protein/lane), in an astrocyte-enriched fraction of hippocampus (20 μg total protein/lane), and in TRPV4-expressing- and uninjected *Xenopus* oocytes (amount of protein corresponding to 0.5 oocyte). The analysis was performed with two sets of individual samples all run as duplicates. Data presented as mean ± SEM and statistical significance determined with a Student's paired t-test. *; *p* < 0.05, ns; not significant.

To investigate a potential role for TRPV4 in regulating cell volume *during a physiologically relevant cell swelling in situ*, independent on an applied osmotic gradient, we approximated a native setting by using acute hippocampal brain slices from rats. Neuronal activity in the brain causes release of K^+^ into the ECS where astrocytes act as K^+^ sinks´ to prevent extracellular K^+^ accumulation and a subsequent widespread depolarization [28,79–82]. In parallel with the management of K^+^, the ECS shrinks [27,28,83-85], a phenomenon primarily attributed to the swelling of nearby astrocytic structures [83,85–86]. Thus electrical stimulation resulting in synaptic activity leads to a brief change in cell volume *without application of an osmotic gradient to the test solution*. This experimental scenario allowed us to test the involvement of TRPV4 in the return of astrocytic cell volume following the stimulus-evoked cell swelling. The relative change in size of the ECS was monitored via a tetramethyl ammonium (TMA^+^)-sensitive microelectrode upon bath application of TMA^+^ (1.5 mM) as described in detail in [27,28]. In brief: Electrical stimulation of the CA1 Schaeffer collaterals of the hippocampus results in a shrinkage of the ECS and thus an increase in concentration of the membrane impermeable TMA^+^. This volume trace then serves as an indirect readout of (astrocytic) cell swelling; see representative traces of stimulus-evoked ECS shrinkage illustrated in (Fig. 3A-C, left). To resolve the involvement of TRPV4 in the volume response, the non-selective TRPV4 inhibitor ruthenium red (RR; 1 µM) was applied to the recording chamber and the volume response recorded after ∼6 min (Fig. 3A). An additional volume response was recorded after another 10 min of inhibitor incubation to ensure that the inhibitor had reached its full effect at the time of recording (for all test conditions). Inhibition of TRPV4 caused a slight increase in peak ECS shrinkage (to 105.5 ± 1.4% of control, *p* < 0.05, n = 7 slices from 4 rats) (Fig. 3A, right) but did not affect the regulatory volume decrease as the rate of return to baseline volume was undisturbed (the decay constant was 104.7 ± 2.4% of control, p = 0.10), see (Fig. 3A, right). In a similar fashion, the more specific TRPV4 inhibitor HC067047 (1 µM) affected the peak ECS shrinkage (116.9 ± 4.7% of control, *p* < 0.05, n = 5 slices from 3 rats), (Fig. 3B, right) but did not influence the return of the ECS back to baseline as evaluated from the decay constant (106.5 ± 7.5% of control, p = 0.43) (Fig. 3B, right). Activation of TRPV4 with the agonist GSK1016790A (GSK101; 100 nM, Fig. 3C) had no significant effect on the ECS dynamics in hippocampal brain slices (peak amplitude amounting to 115.0 ± 6.4% of control, p = 0.13 (Fig. 3C, right), and the decay constant to 107.7 ± 9.5%, n = 6 slices from 4 rats (Fig. 3C, right)). Taken together, there appears to be little, if any, effect on astrocytic regulatory volume mechanisms of either blocking or activating TRPV4 during stimulus-evoked ECS shrinkage in acute hippocampal slices from rats. Notably, during the experimental paradigm, no osmotic gradients were introduced and the induced cell volume change occurred solely as the result of synaptic activity. TRPV4 expression in hippocampal astrocytes was previously demonstrated by immunocytochemistry [11,87–92]. However, the cortical TRPV4 expression was later shown confined to a subset (∼30%) of astrocytes [93] and the astrocytic TRPV4 transcript level reported to be fairly low [94,95]. To determine TRPV4 expression in rat hippocampus, we performed Western blotting analysis on homogenates obtained from rat hippocampi (P21) and from astrocyte-enriched fractions (P20-23) [96]. Efficient antibody recognition was verified in membrane preparations from uninjected and rat TRPV4-expressing *Xenopus laevis* oocytes [9], and GAPDH was used as loading control. Although we did not detect TRPV4 expression in 20 μg loaded protein (Fig. 3G), another research group detected hippocampal TRPV4 expression by Western blot upon loading a 4-fold larger quantity of lysate [88]. The discrepancy between the data obtained by transcriptomics, Western blot, and immunohistochemistry may indicate a low expression of TRPV4 in astrocytes *in situ* and/or in a select subset of astrocytes, as detected in cortex [93].

## Conclusion

Volume regulation is an essential property of all cell types and a range of volume-sensing ion channels and transporters have been revealed and implicated in cell volume regulation throughout the years. Nevertheless, the finer details regarding *exactly* how volume changes occur in physiology, and which mechanisms are involved in returning cell volume to its origin, remain largely unresolved. TRPV4 is a well-established sensor of volume changes, although predominantly during swift cell swelling [9]. Regardless, its implication in the subsequent cell volume regulatory events is less evident, and possibly not even pertinent under more physiological experimental conditions, in which cell swelling is obtained in the absence of externally applied osmotic gradients. For reasons of technical ease, experimenters often introduce such non-physiologically large osmotic gradients to study the ensuing volume-dependent activity of transporters and ion channels. While cell swelling/shrinkage is readily obtained in this manner, mammalian cells hardly experience these conditions *in situ* and we may simply be studying a different rate-limiting step under such circumstances. Cells exposed to an osmotic gradient ought to swell to a similar degree, whether or not an AQP is expressed, and whether or not the osmotic gradient is applied externally or from altered cellular transport activity *in vivo*. However, *the rate* with which it swells will depend on expression of an AQP. Abrupt introduction of a large osmotic gradient will thus allow investigation of cellular responses to a swift cell swelling of a magnitude and speed rarely observed in physiology and most prominently in settings with expression of an AQP. As a range of pathologies are associated with altered fluid dynamics, it is imperative that the research field as a whole obtains better tools as well as understanding of the molecular mechanisms underlying these fascinating volume regulatory properties. Our aim with this follow-up article is to pinpoint a few potential pit-falls along the path to reach this understanding.

## Abbreviations

AQP4: aquaporin 4
ECS: extracellular space
GAPDH: Glyceraldehyde 3-phosphate dehydrogenase
GSK101: GSK1016790A
OTRPC4: *O*SM9-like *t*ransient *r*eceptor *p*otential *c*hannel, member 4
RR: ruthenium red
SPAK/WNK: SPS1-related proline/alanine-rich kinase/with no lysine
TMA^+^: tetramethyl ammonium
TRPV4: Transient receptor potential vanilloid 4
VR-OAC: *v*anilloid *r*eceptor related and *o*smotically *a*ctivated *c*hannel
